# Increase in Colorectal Surgical Site Infections at Two AdventHealth Central Florida Division Campuses: A Quality Improvement Approach

**DOI:** 10.1017/ash.2025.412

**Published:** 2025-09-24

**Authors:** Heyda Rodriguez De Jesus, Ioana Chirca

**Affiliations:** 1 AdventHealth; 2 AdventHealth

## Abstract

**Background:** In 2024, an increase in colorectal surgical site infections (SSI) was identified at two AdventHealth Central Florida Division campuses. Control charts revealed this as a special cause variation, prompting an in-depth investigation into potential contributing factors and modifiable risks. **Methods:** A detailed review of 14 SSI cases (five at Campus A and nine at Campus B) was conducted. The analysis focused on key perioperative practices, including chlorhexidine gluconate (CHG) application, postoperative blood glucose control, perioperative antibiotic administration, hair clipping techniques, skin preparation protocols, and postoperative temperature monitoring. A secondary analysis was conducted by the surgical team on intraoperative factors and operative techniques. **Results:** The investigation revealed that for both campuses, over 80% of SSIs were linked to defects in perioperative antibiotic administration, postoperative glucose control, and CHG application. At Campus A, primary gaps were identified in postoperative glucose control, perioperative antibiotic administration, and CHG application, while at Campus B, issues stemmed from CHG application, postoperative glucose control, and hair removal practices. **Conclusion:** Corrective actions were recommended to address these gaps, including enhanced pharmacy involvement to ensure timely and appropriate antibiotic administration, standardized processes for glucose monitoring and CHG application, and standardization of adherence to WHO guidelines. A continuous audit system with regular feedback was proposed to monitor compliance and drive further improvements. This analysis also revealed the importance of creating a robust process metric dashboard to monitor and provide the ability to detect variation in perioperative processes that could lead to an increase in SSI. It also highlights how quality improvement science has great applicability in infection prevention and hospital epidemiology. These measures aim to significantly reduce SSI rates and improve patient care.

